# Temperature Shifts for Extraction and Purification of Zygomycetes Chitosan with Dilute Sulfuric Acid

**DOI:** 10.3390/ijms11082976

**Published:** 2010-08-13

**Authors:** Akram Zamani, Lars Edebo, Claes Niklasson, Mohammad J. Taherzadeh

**Affiliations:** 1 School of Engineering, University of Borås, 50190 Borås, Sweden; E-Mail: Mohammad.Taherzadeh@hb.se; 2 Department of Chemical and Biological Engineering, Chalmers University of Technology, 41296 Gothenburg, Sweden; E-Mail: claesn@chalmers.se; 3 Department of Clinical Microbiology, University of Gothenburg, 41346 Gothenburg, Sweden; E-Mail: lars.edebo@microbio.gu.se

**Keywords:** fungal chitosan, phosphate release, Rhizomucor pusillus, sulfuric acid

## Abstract

The temperature-dependent hydrolysis and solubility of chitosan in sulfuric acid solutions offer the possibility for chitosan extraction from zygomycetes mycelia and separation from other cellular ingredients with high purity and high recovery. In this study, *Rhizomucor pusillus* biomass was initially extracted with 0.5 M NaOH at 120 °C for 20 min, leaving an alkali insoluble material (AIM) rich in chitosan. Then, the AIM was subjected to two steps treatment with 72 mM sulfuric acid at (i) room temperature for 10 min followed by (ii) 120 °C for 45 min. During the first step, phosphate of the AIM was released into the acid solution and separated from the chitosan-rich residue by centrifugation. In the second step, the residual AIM was re-suspended in fresh 72 mM sulfuric acid, heated at 120 °C and hot filtered, whereby chitosan was extracted and separated from the hot alkali and acid insoluble material (HAAIM). The chitosan was recovered from the acid solution by precipitation at lowered temperature and raised pH to 8–10. The treatment resulted in 0.34 g chitosan and 0.16 g HAAIM from each gram AIM. At the start, the AIM contained at least 17% phosphate, whereas after the purification, the corresponding phosphate content of the obtained chitosan was just 1%. The purity of this chitosan was higher than 83%. The AIM subjected directly to the treatment with hot sulfuric acid (at 120 °C for 45 min) resulted in a chitosan with a phosphate impurity of 18.5%.

## 1. Introduction

Chitosan is a linear cationic polysaccharide that is nowadays mainly produced by chemical deacetylation of chitin from shellfish wastes. An alternative source for this biopolymer is the cell wall of zygomycetes fungi, which contain up to 50% chitosan [1,2]. In this class of fungi, chitosan is a major part of the cell wall, in addition to other components such as phosphates and proteins [2]. The solubility of chitosan in acidic solutions such as acetic acid offers the possibility for its purification from the fungal cell wall [3–7]. However, acetic acid is not effective in extracting the whole chitosan present in the cell wall [7]. On the other hand, a method has recently been developed for extraction of chitosan from zygomycetes cell wall, which is based on the temperature-dependent solubility of chitosan in dilute sulfuric acid solutions. This temperature-dependent solubility is a well known and unique property of chitosan, which is not shared with other components of the cell [7]. In this process, the fungal cell wall is treated with dilute sulfuric acid at e.g., 120 °C for 20 min in an autoclave to dissolve the chitosan. Then, the extracted chitosan is recovered from the acid solution by precipitation at lower temperatures. This new extraction method was able to extract chitosan completely from fungal cell wall, and had a higher recovery compared to the acetic acid extraction process [7].

The extraction and precipitation of pure shellfish chitosan with dilute-sulfuric acid may be accompanied by partial depolymerization of chitosan as well as reaction of chitosan with sulfate ions, such that by cooling the solution, the product precipitates and becomes insoluble in acetic acid solutions [8]. However, raising the pH of chitosan-sulfate solution to 8–10 was shown to result in soluble chitosan in acetic acid [8]. It probably means that a pH-increasing step is also needed in the sulfuric-acid extraction process for production of fungal chitosan. We examined the effect of this step on the solubility of fungal chitosan in acetic acid solutions. The results indicated that the extract precipitated in cold acid showed characteristics different to pure chitosan, since it was not soluble in acetic acid solutions even after increasing the pH to 8–10. Hence, further investigations were required in order to raise the purity of the fungal chitosan extracted with hot sulfuric acid and precipitated in the cold one. Furthermore, the extraction process has to take into account that depolymerization of chitosan occurs during the contact with hot sulfuric acid which may decrease the recovery of pure chitosan from the sulfuric acid solution [8]. These facts became the basis of the present investigation, which aimed at minimizing the hydrolysis and loss of fungal chitosan as well as its possible contaminations during the extraction with sulfuric acid.

## 2. Results

### 2.1. Treatment Durations and Solubilizationof Alkali Insoluble Material (AIM)

#### 2.1.1. Precipitation with NaOH at pH 8–10 in an Ice Bath (CAlP)

**Figure 1:**
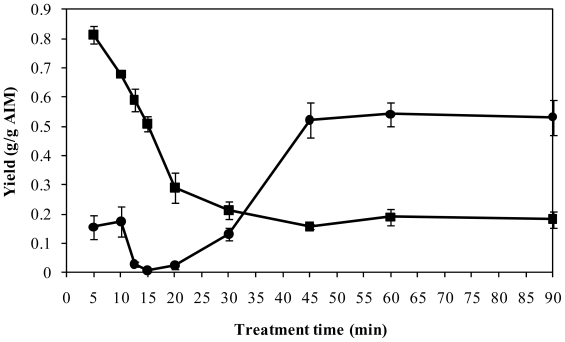
The profile of CAlP (●) and HAAIM (■) production after treatment of AIM with 72 mM sulfuric acid at 120 °C for 5–90 min.

Alkali insoluble material (AIM) of fungal biomass was treated with 72 mM sulfuric acid at 120 °C for 5–90 min and the yields of cold alkali precipitate (CAlP) and hot alkali and acid insoluble material (HAAIM) were measured (Figure 1). When the AIM was treated with the hot acid for 5 min, 15.5 and 80% of AIM were recovered as CAlP and HAAIM, respectively. By increasing the treatment time to 10 min, the yield of HAAIM decreased to 68%, while the yield of CAlP increased to 17.5%. However, further increasing the treating time to 15 min resulted in a sharp decrease in CAlP yield, and also continuous reduction in the yield of HAAIM with almost a constant rate (Figure 1). For treatment times between 15 and 45 min, the CAlP yield increased from 2 to 52% of AIM, while the HAAIM yield decreased further from 29 to 16% (Figure 1). The treatment periods longer than 45 min and up to 90 min had practically no effect on the yield of neither CAlP nor HAAIM (Figure 1).

#### 2.1.2. Precipitation without pH Alteration in an Ice Bath

**Figure 2:**
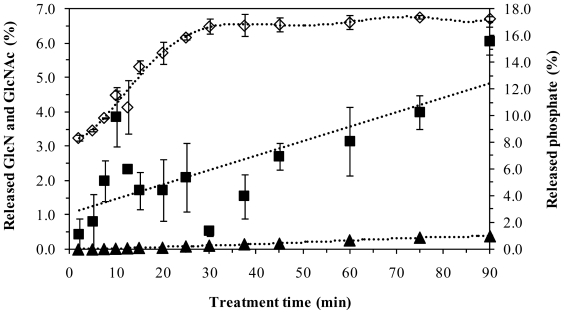
Release of soluble GlcN (■), GlcNAc (▴) and phosphate (⋄) in treating AIM with 72 mM sulfuric acid at 120 °C for 2–90 min.

After the treatment of AIM with sulfuric acid at 120 °C for different times and separation of HAAIM by filtration at high temperature, the temperature was lowered in an ice bath without neutralization with NaOH, which caused precipitation (formation of cold precipitated material or CPM). After centrifugation, the sulfuric acid solutions were analyzed for GlcN and acetic acid. Acetic acid represents the deacetylated GlcNAc and GlcN represents both free and polymer hexosamines which were convereted to anhydromanose via the reaction with nitrous acid and measured by a colorimetric method [9,10]. The results indicated that less than 4% of AIM appeared as soluble GlcN after precipitation in an ice bath when the sulfuric acid treatment was performed for less than 60 min. This solubilization increased to 6.1% by increasing the treatment time to 90 min (Figure 2). Furthermore, the deacetylation of GlcNAc corresponded to less than 0.5% of the AIM after acid treatment for 2–90 min (Figure 2). In contrast, the treatment with hot sulfuric acid resulted in rapid release of phosphate ions, *i.e.*, more than 8% of the AIM within just 2 min. This releasing of phosphate increased continuously to 17% within 30 min, where it was almost constant (Figure 2).

### 2.2. Composition of CAlP

**Figure 3:**
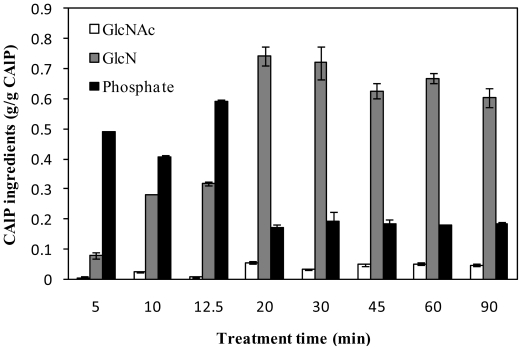
GlcNAc, GlcN and phosphate contents of CAlP obtained after treatment of AIM with 72 mM sulfuric acid at 120 °C for 5–90 min.

The glucosamine (GlcN), *N*-acetyl glucosamine (GlcNAc) and phosphate contents of CAlP obtained from AIM after different treating times are presented in Figure 3. Interestingly, with short extraction times (*i.e.*, less than 20 min) the major ingredient of the CAlP appeared as phosphate (up to 60% of CAlP). After 5 min treatment, the precipitate contained less than 10% of GlcN and GlcNAc, which are the building blocks of chitosan chains. This proportion increased to 33% with a 12.5 min treatment time. Treatment times longer than 20 min resulted in GlcN and GlcNAc as the major components (65–80%) while phosphate made up 17–19% of the CAlP. Consequently, chitosan-rich CAlP was not obtained when the time of treatment was less than 20 min with phosphate as the major impurity (Figure 3).

### 2.3. Purification of CAlP and Production of Phosphate-Free Fungal Chitosan

As shown above, chitosan-rich CAlP can be obtained by 20–90 min treatment of AIM with hot sulfuric acid. The yield of CAlP was shown to increase by increasing the time of the process from 20 to 45 min. No significant increase of the yield was obtained after longer periods of treatment. With 45 min treatment, the chitosan loss (*i.e.*, sum of GlcN and GlcNAc) was less than 3% (Figures 1–3). Therefore, 45 min was chosen as the optimum treatment time in which CAlP with a high yield and high chitosan content was obtained.

**Figure 4:**
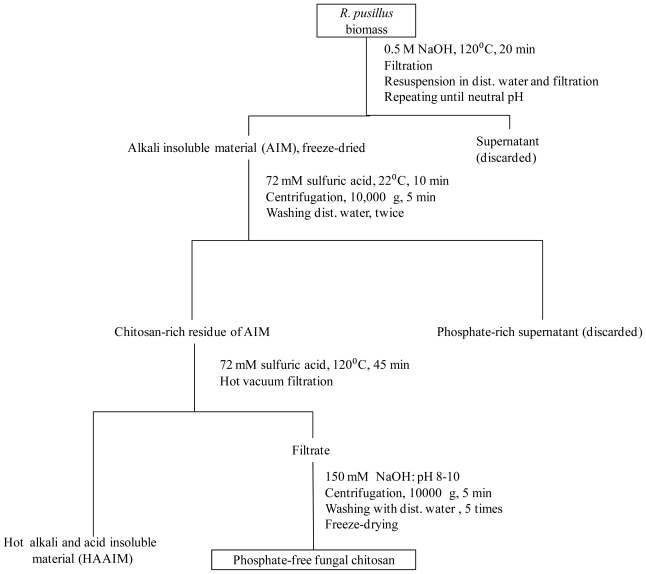
Flow chart of extraction and purification of fungal chitosan by dilute sulfuric acid solution. The phosphate release condition described in this Figure is as for method 1 (presented in Experimental Section), in which AIM is exposed to 72 mM sulfuric acid for 10 min at room temperature.

In spite of significant release of phosphate from AIM in hot sulfuric acid during the process, phosphate was the major impurity of the chitosan-rich CAlP (Figures 2 and 3). More than 75% of the phosphate release occurred in less than 20 min (Figure 2), when the precipitated extract (CAlP) and consequently the chitosan yield, was at its minimum. Therefore, in order to improve the purification process of chitosan, extraction of phosphate before chitosan was attempted by using 72 mM sulfuric acid as a proceeding step (Figure 4).

In these experiments, AIM was suspended in 72 mM sulfuric acid and treated under different conditions including shaking at room temperature, heating at 120 °C, sonication at room temperature or 50 °C and microwave heating. After the pretreatments, the solid residues of AIM were separated from the sulfuric acid solution by centrifugation and washed twice with water to remove all of the released phosphate. Mixing of AIM with pure water instead of sulfuric acid did not result in any phosphate release from AIM. Finally, CAlP was obtained from the residue of AIM within 45 min at 120 °C (Figure 4 and Table 1). The GlcN, GlcNAc, and phosphate contents of the CAlP obtained in this process were also measured (Table 2). When the washing step was performed at room temperature for 10 min, 34% of AIM was recovered as CAlP (Table 1). This CAlP was a chitosan with more than 83% purity (sum of GlcN and GlcNAc content) and just 1% phosphate impurity. Increasing the time of the washing step to 90 min did not improve the purity of CAlP whilst the yield of CAlP was decreased to 29%. Sonication and microwave heating of AIM had neither a significant effect on the purity nor on the yield of CAlP (Tables 1 and 2). However, when the phosphate release was performed at 120 °C, the yield of CAlP was decreased to 21–26%, whereas its purity was not improved significantly (Tables 1 and 2). The yield of HAAIM for all of the pretreatments was 13–16% of AIM (Table 1). Combination of the data presented in Tables 1 and 2 indicates that the yield of GlcN plus GlcNAc recovered as CAlP (g/g AIM), was the highest with 10 min pretreatment at room temperature. Consequently, there is destruction at higher temperatures and with longer pretreatment times (Table 2).

In summary, fungal chitosan with a high purity and yield was obtained from AIM by a two-step dilute-sulfuric acid treatment according to Figure 4. The chitosan that was obtained in this method showed the same characteristics as pure chitosan in terms of solubility in acetic acid solutions.

## 3. Discussion

With advances in fermentation technology, production of chitosan from the cell wall of zygomycetes fungi received increasing interest in recent years [2–5,7,11,12]. In this work, the recently developed sulfuric acid extraction method [7] was improved and optimized, such that chitosan was extracted with higher purity.

It was previously reported that the pure commercial shellfish chitosan was dissolved in 72 mM sulfuric acid solution at 120 **°**C in less than 5 min. By keeping this chitosan in hot sulfuric acid solution, the recovery of chitosan was decreased significantly [8]. However, under identical conditions, the fungal chitosan was not extracted significantly until the time of the process was extended to 45 min (Figure 1). During this time, chitosan hydrolysis did not occur considerably, and the loss of chitosan was less than 3% of the AIM (Figure 2). Resistance toward extraction of fungal chitosan might be due to presence of a complex of chitosan with other ingredients of the cell wall, such as phosphate, which may protect the chitosan from dissolution in acids [1,2,7]. Our results show that during the treatment of chitosan with hot sulfuric acid, chitosan was not extracted significantly before the release of phosphate. This phenomenon might be another evidence for the presence of a chitosan-phosphate complex in the cell wall [7]. Although the phosphates were released prior to chitosan in hot acid, the results showed that they precipitated together with chitosan by cooling. As shown in Figure 3, the major part of CAlP for short extraction times was phosphate. The precipitated phosphate might be in the form of polyphosphates. For treatment lengths 5–15 min, as the treatment time was increased, the yield of the phosphate-rich CAlP decreased (Figure 1). This is probably due to depolymerization of polyphosphates and formation of soluble phosphates that were not precipitated and recovered as CAlP (Figure 2).

Despite the possible depolymerization of polyphosphates, after long treatment times, phosphate was still the major impurity found in the chitosan-rich CAlP. Therefore, a chitosan-phosphate complex might be formed and precipitated. Hamdine *et al*. [13] reported that cooling the hot solution of chitosan in phosphoric acid (from 80 ºC to room temperature) results in formation of a chitosanphosphoric acid gel. The authors suggested that formation of this gel may be due to screening the charged glucosamine residues of chitosan by a mono-anionic form of phosphoric acid and also intramolecular hydrogen bonding between P=O and the second and third hydroxyl groups of phosphoric acid and non-reactive parts of chitosan [13]. Precipitation of phosphates together with fungal chitosan may pass through the similar mechanisms. When the phosphate was removed from AIM in a preceding washing step in dilute sulfuric acid at room temperature, a phosphate-free fungal chitosan was obtained (Table 2). In this process, release of phosphate from AIM into sulfuric acid solution might be accompanied by substitution of phosphate ions with sulfate ions in the chitosanphosphate complex in AIM.

Cui *et al*. [14] reported that the interaction of dilute-sulfuric acid and chitosan at room temperature undergoes two major reactions which are protonation of amine groups of glucosamine and ionic linking of chitosan with sulfate ions. In the latter step, sulfate ions bridge between two NH_3_ ^+^ groups of chitosan [14] and result in a complex insoluble in acetic acid [8]. In our study, in order to prevent the cross-linking of fungal chitosan with sulfuric acid, chitosan recovery from hot-dilute-sulfuric acid solution was performed by simultaneous cooling and increasing the pH to 8–10 (Figure 4). The pH increase prevented the formation of sulfate cross-linked chitosan [8], which resulted in an acetic acid soluble fungal chitosan.

## 4. Experimental Section

### 4.1. The Fungal Strain and Production of Biomass

*Rhizomucor pusillus* CCUG 11292 (Culture Collection University of Gothenburg, Sweden) was used in this work. The fungus was cultivated in xylose-rich spent sulfite liquor (SSL) from a paper pulp plant (Domsjö AB, Sweden) after production of ethanol with *Saccharomyces cerevisiae* (SSL-AE). The SSL-AE was autoclaved for sterility and diluted with three parts of tap water such that the sugar concentration became 4.2 g/L. Then, 0.015 M NH_4_H_2_PO_4_ and 0.07 M ammonium hydroxide were added to the medium and the cultivation performed in a 70 l air-lift bioreactor at 37 ± 1 °C and pH 6.0 ± 0.3. Twice a day, 85% of the volume was harvested and replaced by fresh medium. The mycelium pellets were harvested on a screen, washed with water and stored at −20 °C until use.

### 4.2. Preparation of Alkali Insoluble Material (AIM)

Wet mycelium of *Rhizomucor pusillus* which contained 18% dry weight was treated with 0.5 M sodium hydroxide solution at 120 °C for 20 min in an autoclave. Alkali insoluble material (AIM) was separated by filtration and washed several times with distilled water until a filtrate with neutral pH was obtained. It resulted into 0.18 g AIM per g biomass. AIM was then freeze-dried, weighed and stored at room temperature until use.

### 4.3. Release and Recovery of Chitosan-Rich Material from AIM

The freeze-dried AIM was powdered in a kitchen mill and mixed with 72 mM sulfuric acid solution in glass bottles (25 mL acid with 0.25 g AIM). The bottles were then sealed and placed in an oil bath at 120 °C for 5–90 min. After the desired period of time, the mixture was vacuum-filtered while it was still hot (around 100 °C). Then, the mixture was cooled on ice, and 30 mL of 150 mM NaOH solution was added to the filtrate to adjust the pH to 8–10 and precipitate the “cold alkali precipitate” (CAlP). The CAlP was then recovered by centrifugation, washed with water until neutral pH, freeze-dried, weighed and stored at room temperature for further analyses. The hot alkali- and acid-insoluble material (HAAIM) that was collected on the filter was also washed with water until neutral pH, freezedried and weighed.

### 4.4. Release of Glucosamine (GlcN), Acetic Acid and Phosphate from AIM

In order to study the progress of chitosan depolymerization and phosphate release during the sulfuric acid treatment, AIM was exposed to 72 mM sulfuric acid (100 mL acid per g AIM) for 2–90 min at 120 °C. After the treatment, a sample was taken from the mixture at high temperature and stored for phosphate analysis (see below). Then, the mixture was cooled down on ice without addition of sodium hydroxide solution. The cold-acid precipitate was sedimented by centrifugation, and the supernatant was collected and kept at 5 °C for GlcN and acetic acid analyses (see below).

### 4.5. Phosphate Removal from AIM prior to Extraction with Hot Sulfuric Acid

Dissolution of the major impurity of AIM, *i.e.*, phosphate, was tested with the following four different methods:

1. Treatment of AIM with sulfuric acid at room temperature (22 °C). In this method, 72 mM sulfuric acid solution was added to AIM (25 mL acid to 0.25 g AIM), and mixed on a shaker (100 rpm) at room temperature for 10 or 90 min. Then, the mixture was centrifuged and the sediment was collected.
2. Sonication of AIM in sulfuric acid solution. A similar mixture of AIM in sulfuric acid was treated in an ultrasonic water bath for 10 min at 50 °C or room temperature. After the treatment, the solid residue of AIM was collected by centrifugation.
3. Treatment of AIM with sulfuric acid at 120 °C. In this method, the treatment was performed at 120 °C in an oil bath for 2.5 or 10 min and the residue of AIM was collected by centrifugation after the mixture was cooled down.
4. Microwave treatment of AIM in sulfuric acid. In this method, the AIM in sulfuric acid solution was heated in a microwave oven for 1.0 min and the AIM residue was collected by centrifugation (after cooling the mixture).

For all these methods, the residue of AIM was collected as sediment by centrifugation at 10,000 ×g and washed twice with water. Then, it was mixed with 25 mL sulfuric acid solution (72 mM) in glass bottles, treated for 45 min at 120 °C in an oil bath, hot filtered, CAlP was sedimented, collected and dried for further analyses. A flow-diagramof the process is shownin Figure 4.

### 4.6. Determination of Glucosamine (GlcN) and *N*-Acetyl Glucosamine (GlcNAc)

The GlcN and GlcNAc concentrations in the “solid” materials were measured according to a previous report [10]. Briefly, the samples were hydrolyzed into anhydromannose and acetic acid in two steps of hydrolysis with 13 M sulfuric acid at room temperature for 90 min, and 0.45 M sulfuric acid at 120 °C for 1 h, followed by degradation with nitrous acid at room temperature. Anhydromannose that represents the sum of GlcN and GlcNAc, was measured by a colorimetric method [9,10], whereas acetic acid that was a marker for GlcNAc was measured by HPLC with an ion exchange Aminex column (HPX-87H, Bio-Rad, Richmond, CA) at 60 °C with 0.6 mL/min 5 mM sulfuric acid as eluent using a UV-Vis detector (Waters 2486, Waters, MA, USA).

For the “liquid” samples, the same procedure as above was followed except that the sulfuric acid hydrolysis steps were omitted and the samples were subjected directly to nitrous acid degradation and HPLC analysis for glucosamine and acetic acid measurements, respectively.

### 4.7. Determination of Phosphate

Phosphate contents of liquid samples were measured by the ammonium molybdate spectrophotometric method according to European standard, ISO6878 [15]. KH_2_PO_4_ was used as a standard reference.

The experiments were performed in duplicate and the results are presented as averages.

## 5. Conclusions

Treatment of cell wall of *Rhizomucor pusillus* with hot-dilute-sulfuric acid and precipitation of the dissolved fraction resulted in extraction and precipitation of chitosan and phosphate together simultaneously. Phosphate-free fungal chitosan was obtained by subsequent extractions of the cell wall with dilute sulfuric acid at different temperatures. In the first step, phosphates were released and removed from the cell wall at room temperature, while in the second step chitosan was extracted from the phosphate-free cell wall residue by treatment with hot acid. This purified chitosan was recovered from the solution by precipitation at pH 8–10 and lowered temperature.

## Figures and Tables

**Table 1:** The yields of CAlP and HAAIM obtained from AIM after different pretreatments with 72 mM sulfuric acid and washing followed by 45 min treatment with 72 mM sulfuric acid at 120 ºC.

Pretreatment method	CalP (g/gAIM)	HAAIM (g/gAIM)
Room temperature, 10 min, mixing in water bath	0.34 ± 0.04	0.16 ± 0.02
Room temperature, 90 min, mixing in water bath	0.29 ± 0.03	0.14 ± 0.01
Room temperature, 10 min, sonication	0.33 ± 0.03	0.14 ± 0.01
50 °C,10 min, sonication	0.30 ± 0.03	0.13 ± 0.01
120 °C, 2.5 min, treatment in oil bath	0.26 ± 0.08	0.14 ± 0.01
120 °C, 10 min, treatment in oil bath	0.21 ± 0.04	0.15 ± 0.01
1 min, treatment in microwave	0.30 ± 0.05	0.14 ± 0.00
Unpurified CAlP	0.52 ± 0.06	0.16 ± 0.01

**Table 2:** GlcN, GlcNAc, and phosphate content of the CAlP prepared from AIM and the yield of (GlcN+GlcNAc) recovered as CAlP after different pretreatments with 72 mM sulfuric acid and washing followed by 45 min treatment with 72 mM sulfuric acid at 120 °C.

	CalP composition (g/gCAlP)				GlcN + GlcNAc Recoveredas CalP (g/gAIM)

Pretreatment method	GlcN	GlcNAc	GlcN + GlcNAc	Phosphate	
Room Temp, 10 min, mixing in water bath	0.767 ± 0.038	0.070 ± 0.004	0.837 ± 0.040	0.010 ± 0.005	0.287
Room Temp, 90 min, mixing in water bath	0.783 ± 0.023	0.069 ± 0.002	0.851 ± 0.022	0.009 ± 0.003	0.244
Room Temp, 10 min, sonication	0.819 ± 0.055	0.067 ± 0.004	0.887 ± 0.055	0.006 ± 0.002	0.295
50°C, 10 min, sonication	0.783 ± 0.040	0.065 ± 0.002	0.848 ± 0.039	0.006 ± 0.004	0.255
120 °C, 2.5 min, treatment in oil bath	0.748 ± 0.031	0.065 ± 0.002	0.813 ± 0.032	0.012 ± 0.005	0.215
120 °C, 10 min, treatment in oil bath	0.717 ± 0.071	0.057 ± 0.000	0.774 ± 0.071	0.010 ± 0.000	0.161
1 min, treatment in microwave	0.815 ± 0.108	0.061 ± 0.002	0.876 ± 0.100	0.007 ± 0.006	0.260
Unpurified CAlP	0.626 ± 0.004	0.049 ± 0.001	0.675 ± 0.005	0.185 ± 0.006	0.351

## Abbreviations

AIM: Alkali insoluble material
HAAIM: Hot alkali and acid insoluble material
GlcN: Glucosamine
GlcNAc: N-acetyl glucosamine
CAlP: Cold alkali precipitate
CPM: Cold precipitated material
